# Gut Microbiota Aberration in Patients of Systemic Sclerosis and Bleomycin-Induced Mice Model

**DOI:** 10.3389/fcimb.2021.647201

**Published:** 2021-05-28

**Authors:** Jungen Tang, Xin Zhou, Xuefen Wu, Shengyan Lin, Bingxia Ming, Jixin Zhong, Baoju Wang, Lingli Dong

**Affiliations:** ^1^ Department of Rheumatology and Immunology, Tongji Hospital, Tongji Medical College, Huazhong University of Science and Technology, Wuhan, China; ^2^ Department of Infectious Disease, Union Hospital, Tongji Medical College, Huazhong University of Science and Technology, Wuhan, China

**Keywords:** SSc, BLM-induced mice model, gut microbiota, 16S rRNA, microbiota aberration

## Abstract

Systemic sclerosis (SSc) is an immune-mediated systemic autoimmune disease with unknown etiology, which has high morbidity and mortality. Current treatments to dispose of this disorder are limited. And there are still no ideal animal models that can fully replicate the four basic pathophysiological features of SSc, including vascular lesions, fibrosis, inflammation, and autoimmunity, let alone animal models specifically designed to study gastrointestinal lesions. It’s essential to seek and establish appropriate animal models to explore the role of gut microbiota in the pathogenesis of SSc. In this study, we found similar gut microbiota aberration in patients of SSc and bleomycin (BLM)-induced mice model through 16S rRNA gene sequencing. In terms of phylum-level differences, the relative abundance of Bacteroidetes was significantly decreased and Firmicutes increased in the SSc patients and the mice. Notably, the genera of Lactobacillus, commonly used as a probiotic additive, was also elevated in SSc patients and BLM mice, which was consistent with a few of studies. Therefore, the model can likely mimic the pathological changes of gut microbiota in patients with SSc, which may offer an important potential platform for the in-depth understanding of gut microbiota aberration in patients with SSc and to devise potential disease-modifying treatments.

## Introduction

Systemic sclerosis (SSc), also named scleroderma, is an immune-mediated systemic autoimmune disease with unknown etiology. As a rare disease, it has high morbidity and mortality. The main clinical manifestations include skin fibrosis (increased tension), internal organs lesions (lung, kidney, gastrointestinal tract, etc.), and vascular lesions (Denton and Khanna, 2017). Among the organs involved, the gastrointestinal tract (GIT) is the most common one only second to the skin. More than 90% of patients have GIT involvement, which is characterized by dysphagia, esophageal reflux, vomiting, constipation, and abdominal pain (Emmanuel, 2016). The current treatments to dispose of this disorder are limited, mainly adopting corresponding individualized methods to different clinical symptoms. Unfortunately, there are still no ideal animal models that can fully replicate the four basic pathophysiological features of SSc, including vascular lesions, fibrosis, inflammation, and autoimmunity (Asano, 2016), let alone animal models specifically designed to study gastrointestinal lesions. Bleomycin (BLM)-induced SSc model is a widely used inducible animal model for preclinical studies of evaluating anti-inflammatory and anti-fibrotic therapies. Although BLM-induced skin fibrosis is limited to the site of injection, systemic inflammation and autoimmune response also existed in this model, such as damaged lung architecture, the production of anti-nuclear antibodies (anti-Scl-70, anti-U1RNP et al.) (Beyer et al., 2010).

The gut microbiota, harboring most of our microbial population, is an extremely complex community and is considered a human organ or “second genome” for its important role. It is not only related to innate immunity but also adaptive immunity, which has been verified to play an important role in shaping and educating the human immune system (Honda and Littman, 2016; Thaiss et al., 2016). Besides, the metabolites from microbes are also involved in the interplay with immunological processes (Rooks and Garrett, 2016). These strongly indicate that gut microbiota aberration may play a crucial role in the pathogenesis of human diseases. Indeed, the gut microbiota is involved in a variety of autoimmune diseases, such as systemic lupus erythematosus (SLE), type I diabetes, rheumatoid arthritis (RA), primary Sjögren’s syndrome (pSS), and inflammatory bowel disease (Hevia et al., 2014; Alkanani et al., 2015; Zhang et al., 2015; Mandl et al., 2017; Lloyd-Price et al., 2019). Numerous studies have shown gut microbiota differences existed in SSc patients compared to healthy controls, especially in the lower GIT (Volkmann et al., 2016; Patrone et al., 2017; Volkmann et al., 2017; Bellocchi et al., 2018; Bellocchi and Volkmann, 2018). The main common feature was that the levels of the commensal genera decreased while the pathobiont genera increased. Notably, lactobacillus, one of the commensal genera, was significantly augmented in SSc subjects than that in healthy controls, which have been used as supplementary probiotics. Whether the gut microbiota aberration is a result of SSc or a cause remains unclear, and the abnormal change of the members of the lactobacillus genus and its precise role in SSc are also unknown. Therefore, it is essential to seek and establish appropriate animal models to explore the role of gut microbiota in the pathogenesis of SSc.

## Methods

### Patients

The study included consecutive patients admitted to the Department of Rheumatology and Immunology, Tongji Hospital, Tongji Medical College, Huazhong University of Science and Technology, Wuhan, Hubei, China from June 2019 to December 2019, fulfilling the American Congress of Rheumatology/European League Against Rheumatism (ACR/EULAR) 2013 diagnosis criteria for SSc (van den Hoogen et al., 2013). Eventually, five patients were diagnosed initially without administration of steroids or immunosuppressive drugs or any other medical treatments influencing intestinal microbio and ten age and gender-matched healthy controls are involved. All participants were free of infections, application of antibiotics, and probiotics during the three months before the start of the study and were able to provide fresh feces. The patients defined as gastrointestinal tract involvement (GI+) were based on the assessment of the UCLA SCTC GIT 2.0 instrument (Khanna et al., 2009). This study was approved by the Ethics Committee of Tongji Medical College of Huazhong University of Science and Technology with batch number 2019-S1159.

### Mouse Model

In this study, C57BL/6 mice aged 6-8 weeks were purchased from Hunan SJA Laboratory Animal Co., Ltd and reared in SPF (Specific Pathogen Free) class experimental animal center, Tongji Hospital Affiliated to Tongji Medical College of Huazhong University of Science and Technology, with constant temperature and normal diet. BLM was used to induce skin fibrosis in C57BL/6 mice, and BLM (100ul, 1mg/ml) was injected subcutaneously on the upper back of the mice once a day for 3 weeks (Avouac, 2014). The control group was injected with the same amount of sterile phosphate buffered solution at the same location. The skin changes at the subcutaneous injection site of mice were observed daily and the weight changes of mice were monitored. In the fourth week after the modeling, the feces of the mice were collected and stored in a sterile cryopreserved tube at -80°C.

### Fecal DNA Amplification and 16S rRNA Gene Sequencing

A complete collection of stool samples was transported through dry ice for 16S rRNA gene sequencing. Bacterial DNA was extracted from fecal samples using the E.Z.N.A.^®^ soil DNA Kit (Omega Bio-tek, Norcross, GA, U.S.) according to the manufacturer’s guidelines. After obtaining bacterial DNA from fecal samples, they were taken into the centrifuge tube and were diluted with sterile water to 1ng/μl as the reaction substrate. Based on the selection of sequencing regions, the v3-v4 variable region of bacterial 16S rRNA was amplified with the primer pairs 338F (5’- ACTCCTACGGGAGGCAGCAG-3’) and 806R (5’- GGACTACHVGGGTWTCTAAT-3’) using a thermocycler PCR system (GeneAmp 9700; ABI, Carlsbad, CA, USA). Purified amplicons were pooled in equimolar concentrations and paired-end sequenced (2×300) on an Illumina MiSeq platform (Illumina, San Diego, USA) according to the standard protocols in Shanghai Majorbio Bio-pharm Technology Co., Ltd.

### Bioinformatic Analysis of 16S rRNA Gene Sequencing

OTU (Operational Taxonomic Units) is a uniform symbol artificially set up for a Taxonomic unit (strains, genera, species, groups, etc.) in phylogenetic or population genetics studies for the convenience of analysis. Cluster is needed to understand the number information of bacteria species, bacteria genera and so on in the sequencing results of a sample. By clustering, the sequences are divided into subunits according to their similarity to each other, and one subunit is an OTU. According to different similarity levels, all sequences can be divided into OTUs, and the bioinformatics statistical analysis is usually carried out for OTUs at 97% similarity level. The raw fastq files were quality-filtered by Trimmomatic and merged by FLASH refer to the following criteria:(i) Set a 50bp window. If the average quality value in the window is lower than 20, cut back bases from the beginning of the window, filter reads below 50bp after quality control, and remove reads containing N bases; (ii) According to the overlapping relationship between sequencing reads, the pairs of reads are merged into a sequence with a minimum overlap length of 10bp; (iii) The maximum mismatch ratio allowed in the overlap area of the spliced sequence is 0.2, and the non-conforming sequence is screened; (iiii) Distinguish the samples according to the barcode and primers at both ends of the sequence, and adjust the sequence direction. The number of mismatches allowed by the barcode is 0, and the maximum number of mismatches is 2. For optimized sequences, extract non-repetitive sequences and remove single sequences that are not repeated. Perform OTU clustering on non-repetitive sequences according to 97% similarity cutoff using Usearch (vsesion 7.0 http://drive5.com/uparse/), and remove chimeras during the clustering process to obtain OTU representative sequences. The database used for comparison is Silva (Release132 http://www.arb-silva.de). The data were analyzed on the free online platform of Majorbio Cloud Platform (www.majorbio.com).

## Results

### Basic Information of the Study Subjects

**Figure 1:**
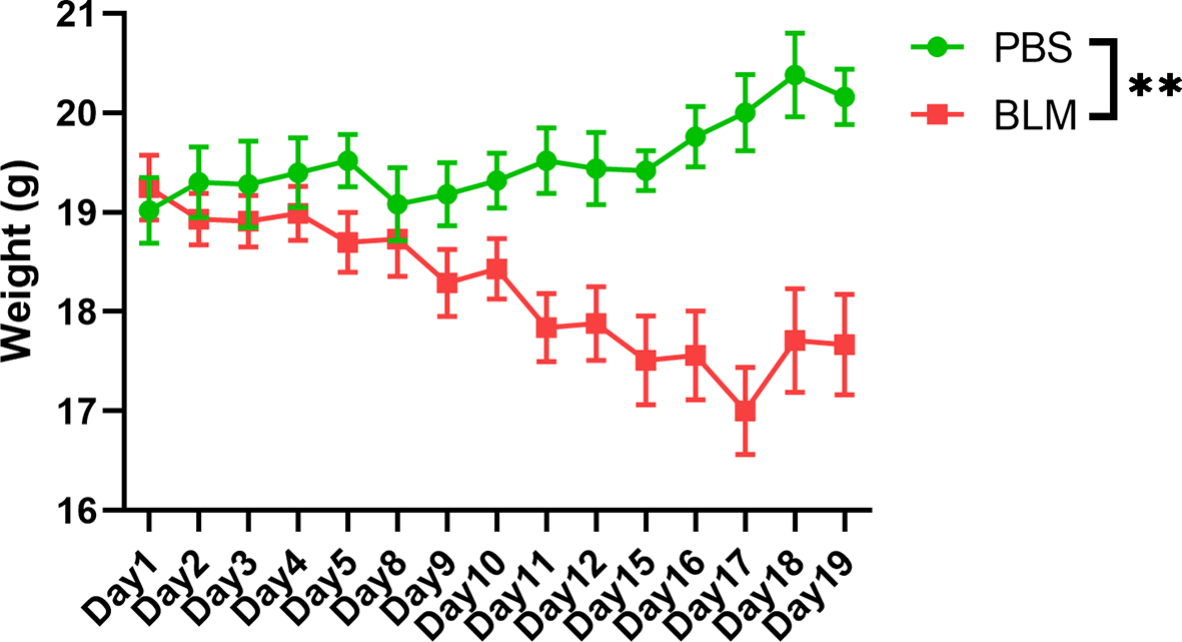
Weight change of the BLM-induced group and the PBS group. The green line represents the physiological changes of bodyweight in PBS group. The red line represents the weight change of BLM group with prolonged induction time. **P < 0.01.

Demographic and clinical parameters of the five enrolled SSc patients and ten health controls are summarized in **Table 1**. SSc patients had a mean age of 45 years and a mean disease duration of 0.97 years, and three of the patients were female. All of them were antinuclear antibody positive (ANA+); Four (80%) were anti-Scl-70 antibody positive(anti-Scl-70+). According to the instrument of UCLA SCTC GIT 2.0, two of the patients were GI+. Healthy controls including six males and four females that have a mean age of 45.60 years. There were no statistical differences in gender and age composition between the healthy controls and the patients. However, the body mass index (BMI) in health controls (23.30 ± 2.75) was significantly higher than that in SSc patients (19.77 ± 2.54). In this study, BLM was used to induce C57BL/6 mouse skin fibrosis model. The results showed that with the prolongation of BLM injection, the skin elasticity of the injection site gradually decreased, the skin toughness gradually hardened, and the skin thickness gradually increased. However, the skin of the PBS control group was normal, with good elasticity and no corresponding changes. The weight monitoring results showed that the bodyweight of BLM-induced mice decreased gradually with the extension of the administration time, while the bodyweight of PBS group mice increased naturally with the growth. No mice died in either group. Model induction was verified by hematoxylin-eosin (HE) pathological staining and hydroxyproline content determination (data not shown). The weight changes of the two groups were shown in **Figure 1**.

**Table 1:** Characteristics of SSc patients and healthy subjects.

Parameters	Healthy controls (n=10)	SSc (n=5)	P
Age, mean ± sd years	45.60 ± 8.73	45 ± 10.79	0.92
Mean disease duration(range) years	N/A	0.97 (0.33-2)	N/A
Male/Female	6/4	2/3	0.61
BMI	23.30 ± 2.75	19.77 ± 2.54	0.04
ANA+	N/A	5	N/A
anti-Scl-70+	N/A	4	N/A
GI+	N/A	2	N/A

BMI, body mass index; ANA+, antinuclear antibody positive; anti-Scl-70+, anti-Scl-70 antibody positive. P, comparison of SSc with health controls; N/A, Not available.

### Microbial Profiling of the Study Subjects

#### Sample Size and Sequencing Depth

A total of 19,329,318 high quality bases were acquired from the 15 human stool samples with average 46,629 sequences for each sample. After sequence extraction and specific species screening, 968 OTUs were determined (with 97% similarity). While 21,030,397 bases were acquired from the 15 mouse stool samples. And averaged 49,903 sequences for each sample and 965 OTUs were acquired. The Shannon-rarefaction curve (**Figure S1**) was applied to judge whether the sequencing data is sufficient and suitable according to whether the curve reaches a gentle level.

#### Alpha Diversity Analysis and Microbiota Compositional Changes

**Figure 2:**
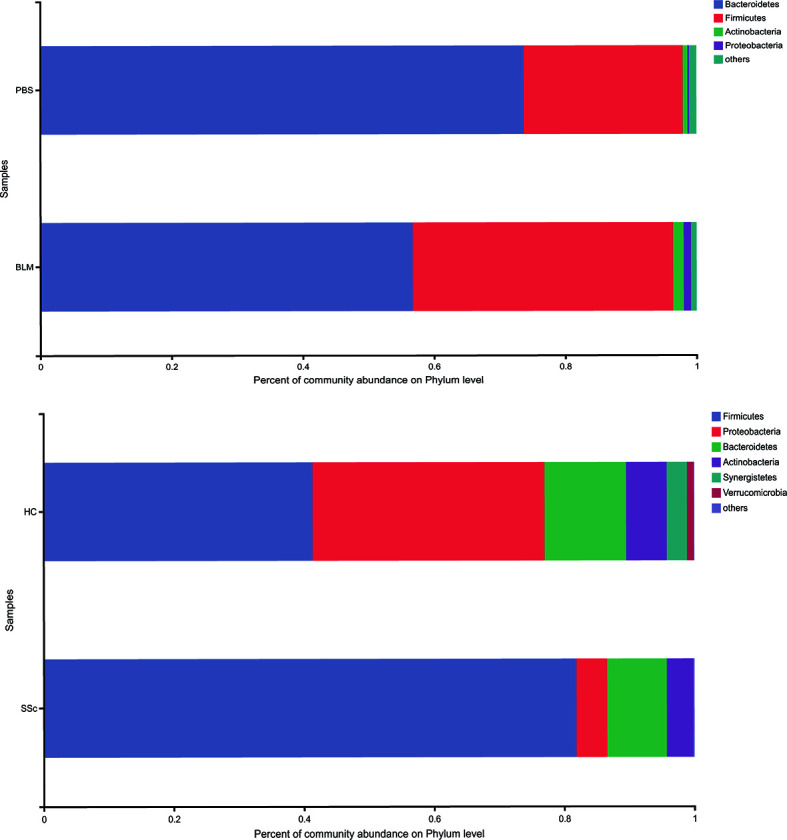
Microorganism community structures at the phylum level. Different colors represent different bacteria. The abundance of Firmicutes was increased while Bacteroidetes were decreased in SSc patients and BLM-induced mice.

**Figure 3:**
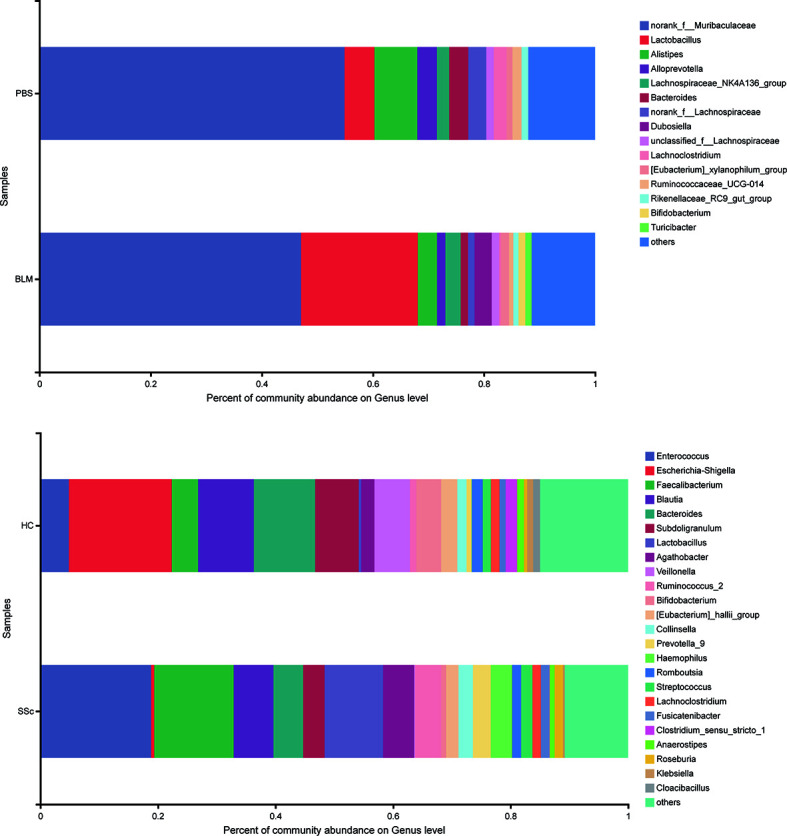
Microorganism community structures at the genus level. Different colors represent different bacteria. The levels of Lactobacillus in the feces of SSc patients increased compared with the healthy control group at the taxonomic level of the genus, which also happened in the mouse model. And the levels of Bacteroides decreased in the SSc patients and BLM-induced mice.

Alpha diversity refers to the diversity within a specific region or ecosystem, which is measured by indicators such as Chao, Shannon, Ace, and Simpson. The diversity of species and other information can be obtained by observing various index values. In the experiment, the statistical T-test method was used to detect whether there was a significant difference in index values between the two groups. After analysis, there was no statistically significant difference in diversity between the two groups in either mice or humans (**Figure S2**). Strikingly, the differences between the two species’ groups have similar changing trends on index average (**Table 2**). The fecal microbiota compositions in the experimental groups of subjects are presented at the phylum level in **Figure 2**. A dominance of Firmicutes, followed by Proteobacteria, Actinobacteria, and Bacteroidetes was found, and the abundance of Firmicutes was increased while Bacteroidetes were decreased in SSc patients compared to the healthy control. In light of recent studies, the Firmicutes-to-Bacteroidetes ratio is abnormal in several autoimmune diseases, such as SLE, pSS, and RA, with varying conclusions (Hevia et al., 2014; Siddiqui et al., 2016; Rogier et al., 2017). Although the dominant species of mice were slightly different from humans, Firmicutes and Bacteroidetes also showed the same variation between the BLM group and the Control group (**Figure 3**). Meanwhile, the levels of Lactobacillus in the feces of SSc patients increased compared with the healthy control group at the taxonomic level of the genus, which also happened in the mouse model. And the levels of Bacteroides decreased in the SSc patients and BLM-induced mice.

**Table 2:** The mean alpha richness and diversity indexes of the samples on OTU level.

Sample	Species richness		Species diversity	
	Ace	Chao	Shannon	Simpson
**PBS (n=5)**	399.193	409.759	4.125 ↑	0.034
**BLM (n=10)**	417.735 ↑	421.874 ↑	4.045	0.047 ↑
**HC (n=10)**	195.317	178.192	2.548 ↑	0.199
**SSc (n=5)**	254.781 ↑	239.230 ↑	2.463	0.282 ↑

Species richness: Ace and Chao indices reflect the richness of microbial community. Species diversity: Shannon and Simpson indexes reflect the diversity of microbial communities. ↑, The average of the indexes has the similar changing trend.

#### Beta-Diversity Analyses

**Figure 4:**
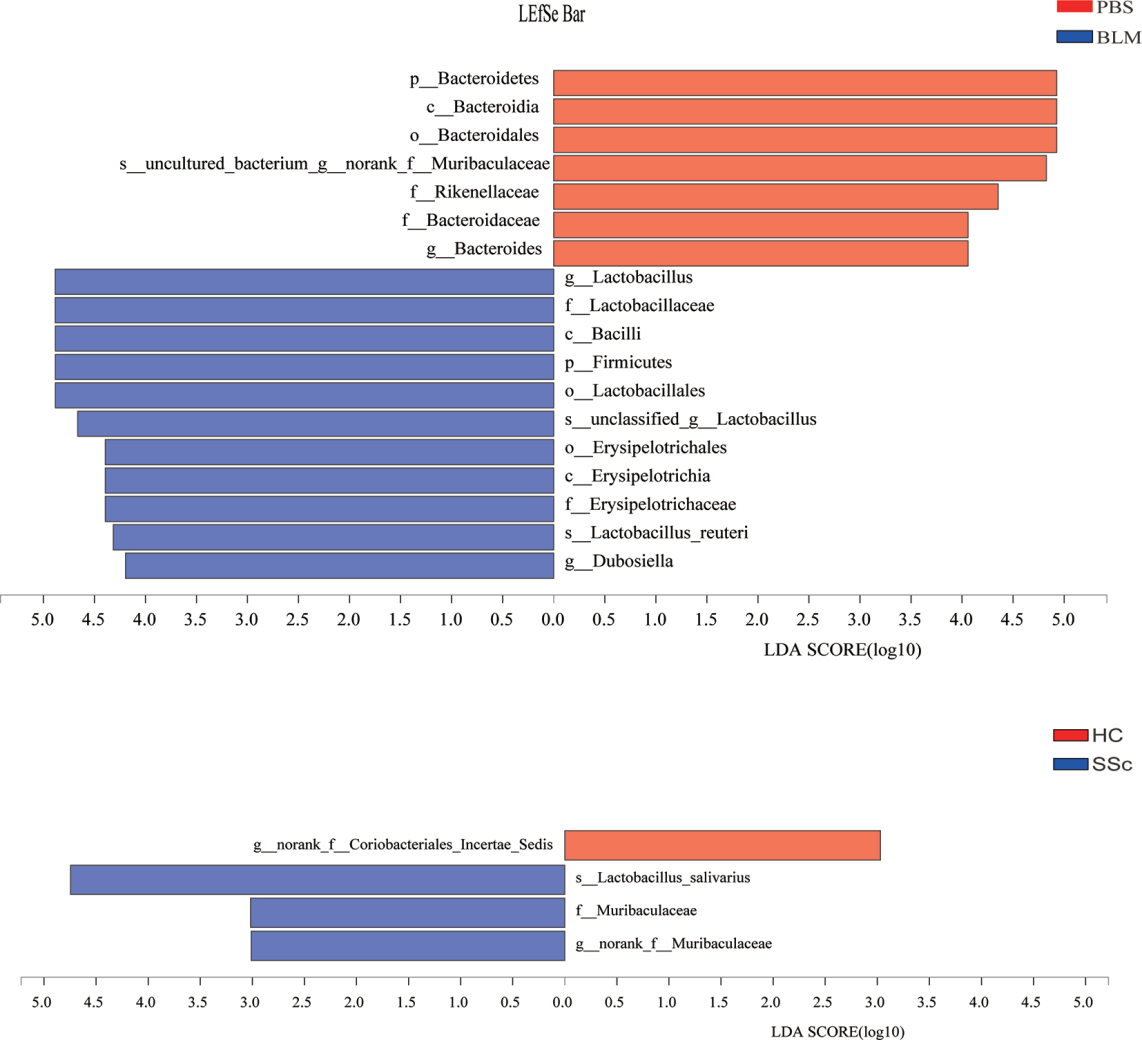
Different gut microbiota analysis using Lefse software. Taxa enriched in the SSc patients and BLM-induced mice are indicated by a negative LDA score (blue), and taxa enriched in healthy individuals and PBS mice have a positive score (red). Only taxa meeting an LDA significant threshold of 3.0 are shown. LEfSe analysis showed that Lactobacillus and Firmicutes were significantly enriched in BLM fecal samples, while Bateroidales were enriched in the control group. Significant accumulation of Lactobacillus_salivarius in SSc feces. Different colors represent different groups.

LEfSe is a kind of software discovering high-dimensional biological markers and revealing genomic features, including genes, metabolisms, and taxa, which is used to distinguish two or more biological conditions. LEfSe analysis (LDA threshold is three) showed that Lactobacillus and Firmicutes were significantly enriched in fecal samples of mice from the BLM group, while Bateroidales were enriched in samples from the control group. Significant accumulation of Lactobacillus_salivarius in SSc feces was also found (**Figure 4**).

**Figure 5:**
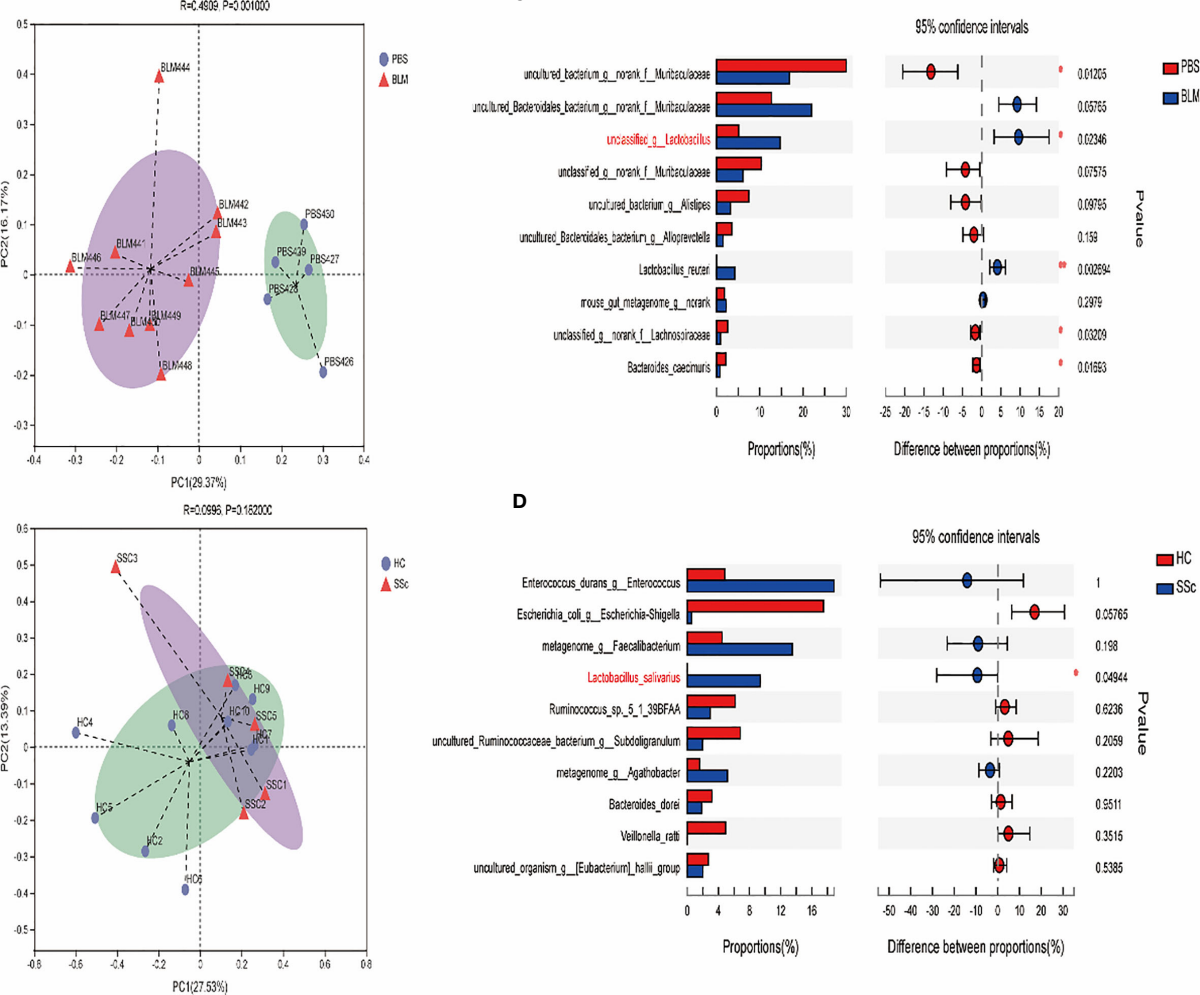
Differences in bacterial composition in the patents and mice. **(A, B)** PCoA analysis showed that the intestinal microbial composition of mice in different treatment groups was significantly different (P=0.001), and the interpretation degree of PC1 and PC2 was 29.37% and 16.17%, respectively. There was no significant difference between SSc patients and healthy controls (P=0.182). **(C, D)** The microbiological groups with significant differences at the species level were revealed by species difference analysis. The results showed that the abundance of unclassified_g_Lactobacillus and Lactobacillus_reuteri in the BLM group was significantly higher than that in the PBS group (P<0.05, P<0.01 Mann-Whitney U test), and the abundance of Lactobacillus_salivarius in SSc patients was significantly higher than that in the control group (P<0.05, Mann-Whitney U Test).

Principal coordinates analysis (PCoA) based on the Bray-Curtis distance was performed to analyze whether the structure of the bacterial community in the gut microbiota differs between the two groups in both humans and mice. The results in **Figure 5** showed that the gut microbial composition of mice in different treatment groups was significantly different (P=0.001), and the interpretation degree of PC1 and PC2 was 29.37% and 16.17%, respectively. However, there was no significant difference between SSc patients and healthy controls (P=0.182). Based on the obtained community abundance data and using strict statistical methods, hypothesis testing was carried out on the species among different groups of microbial communities to evaluate and obtain the species with significant differences between groups. We found that the abundance of unclassified_g_Lactobacillus and Lactobacillus_reuteri in the BLM group was significantly higher than that in the PBS group (P<0.05, P<0.01 Mann-Whitney U test), and the abundance of Lactobacillus_salivarius in SSc patients was also significantly higher than that in the control group (P<0.05, Mann-Whitney U Test).

## Discussion

Systemic sclerosis is a chronic multiple system autoimmune disorder. It involves the GIT in more than 90% of patients, which can extend from the mouth to the anus. After cardio-pulmonary and renal involvement, the GIT involvement is the third most common cause of mortality and the leading cause of morbidity in SSc patients (Hunzelmann et al., 2008). Altered gut microbiota may occur and contribute to the initiation, progression or severity of the disease. Although several studies have shown that the gut microbiota of SSc patients is abnormal compared to that of normal people (Andréasson et al., 2016; Volkmann et al., 2016; Patrone et al., 2017; Bellocchi et al., 2018), it remains unclear whether the changes in the gut microbiota are results of disease or initiating causes. To our knowledge, this is the first study to elevate the gut microbiota profile in an SSc mouse model and compared to that in humans. According to the analogy analysis, it is preliminarily achieved that the BLM-induced SSc model can be used to study the pathogenic mechanism and treatment of the gut microbiota in this disease.

In this study, we found that the BMI of SSc patients was lower than that of healthy controls. Moreover, mice exposed to BLM on local skin also showed weight loss compared with those exposed to PBS. This suggests that the gut microbial and nutrient absorption functions of the mice may be altered, further suggesting whether this aberration can mimic changes in SSc patients. There was no statistically significant difference in alpha richness and diversity indexes between the groups in both mice and humans. But the variation trends of the mean level are consistent. In terms of phylum-level differences, the relative abundance of Bacteroidetes was significantly decreased and Firmicutes increased in the SSc patients. The same thing happened in the BLM-induced mice model. It has been reported that the ratio of Firmicutes to Bacteroidetes may have a critical important effect on human health (Ley et al., 2006). A lower Firmicutes to Bacteroidetes ratio in systemic lupus erythematosus (SLE) patients has been reported (Hevia et al., 2014). Conversely, Firmicutes are increased in rheumatoid arthritis and Sjogren’s syndrome patients (Siddiqui et al., 2016; Rogier et al., 2017). Consisted of the findings of Elizabeth R Volkmann. et al. and Vania Patrone et al. we found this ratio was increased in both SSc patients and BLM mice (Andréasson et al., 2016; Volkmann et al., 2016). When it comes to the lower level taxonomic analysis, both SSc cohorts and BLM mice had significantly lower levels of the commensal genera Bacteroides, which is thought to protect the host from mucosal inflammation and against colonization of pathogenic species. In other chronic inflammatory diseases, a low relative abundance of bacteroides is associated with increased disease activity, such as Crohn’s disease (Schäffler et al., 2016). Notably, the genera of Lactobacillus, commonly used as a probiotic additive, was also elevated in SSc patients and BLM mice, which was consistent with a few studies. And in agreement with Andreasson et al. and Vania Patrone et al., we observe a trend towards a decrease of Bifdobacterium at the genus level in fecal samples from SSc patients (Andréasson et al., 2016; Patrone et al., 2017). But in the study of Elizabeth R Volkmann.et al, the genus was increased in the disorder (Volkmann et al., 2016). The gut microbial composition of mice in different treatment groups was significantly different according to the PCoA analysis. Due to other factors such as living environment, diet, and course of the disease, differences in the fecal microbial composition of SSc patients are not significant. This may also explain the slight variation in genera among different studies to a certain extent. Through analyzing the differences at the species level, the abundance of unclassified_g_Lactobacillus and Lactobacillus_reuteri in the BLM group was significantly higher than that in the PBS group and the abundance of Lactobacillus_salivarius in SSc patients was significantly higher than that in the control group. This would break our traditional understanding of the “protective,” commensal species, which may play different roles in the context of the underlying disease state. This may also explain the probiotic supplements have a limited effect in improving gastrointestinal symptoms in SSc patients in a recent study (Marighela et al., 2019). Due to the lack of in-depth understanding of the mechanism, the treatment of systemic sclerosis gastrointestinal symptoms still relies on symptomatic support therapy. However, patients still need to receive effective treatment to improve their quality of life. A recent study suggests that flora transplantation has the potential effect to improve gastrointestinal symptoms in patients (Fretheim et al., 2020). However, a lack of mechanical understanding and small populations make its application very limited. In a mouse model of SSc associated with anti-topoisomerase-I immunity, early life antibiotic exposure causes intestinal dysbiosis and exacerbates skin and lung pathology (Mehta et al., 2017). In our study, the gut microbiota structures of BLM-induced mice were similar with SSc patients in many aspects, which provided a good reference model.

## Conclusion

In sum, the present study of gut microbiota aberration in patients of systemic sclerosis and BLM-induced mice model is small and requires further confirmation and study. However, the model can likely mimic the pathological changes of gut microbiota in patients with SSc. In the future, this may offer an important potential platform for the in-depth understanding of gut microbiota aberration in patients with SSc and to devise potential disease-modifying treatments for particular strains.

## Data Availability Statement

The datasets presented in this study can be found in online repositories. The names of the repository/repositories and accession number(s) can be found below: NCBI, The BioProject number is PRJNA723505 and the SRA accession number is SRP315740.

## Ethics Statement

The studies involving human participants were reviewed and approved by the Ethics Committee of Tongji Medical College of Huazhong University of Science and Technology (2019-S1159). The patients/participants provided their written informed consent to participate in this study. The animal study was reviewed and approved by the Ethics Committee of Tongji Medical College of Huazhong University of Science and Technology (2019-S1159). Written informed consent was obtained from the owners for the participation of their animals in this study. Written informed consent was obtained from the individual(s) for the publication of any potentially identifiable images or data included in this article.

## Author Contributions

All authors listed have made a substantial, direct, and intellectual contribution to the work and approved it for publication.

## Funding

This work was supported by grants from the National Natural Science Foundation of China (no. 81771754 to LD) and the Tongji Hospital Clinical Research Flagship Program (no. 2019CR206).

## Conflict of Interest

The authors declare that the research was conducted in the absence of any commercial or financial relationships that could be construed as a potential conflict of interest.
