# Supplementary material for: Gut Microbiota Aberration in Patients of Systemic Sclerosis and Bleomycin-Induced Mice Model
Source: Front Cell Infect Microbiol. 2021 May 28;11:647201. doi: 10.3389/fcimb.2021.647201 (PMC8193929; doi:10.3389/fcimb.2021.647201)
Supplement: Supplementary file 1 [file DataSheet_1.doc]

**Supplementary appendix**


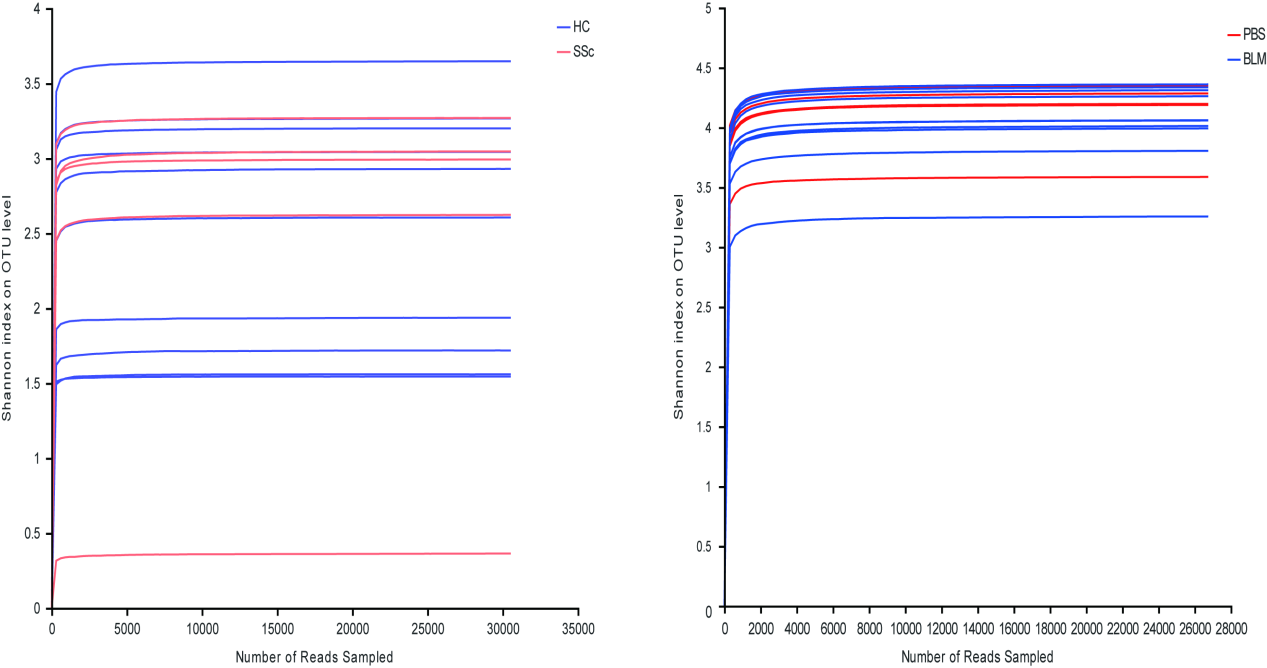


**Supplementary Figure S1.** The Shannon-rarefaction curves. In both human samples and mouse samples, the curves tend to be flat as the number of samples increases,which indicates the sequencing data volume is large enough to reflect the vast majority of microbial diversity information in the samples.


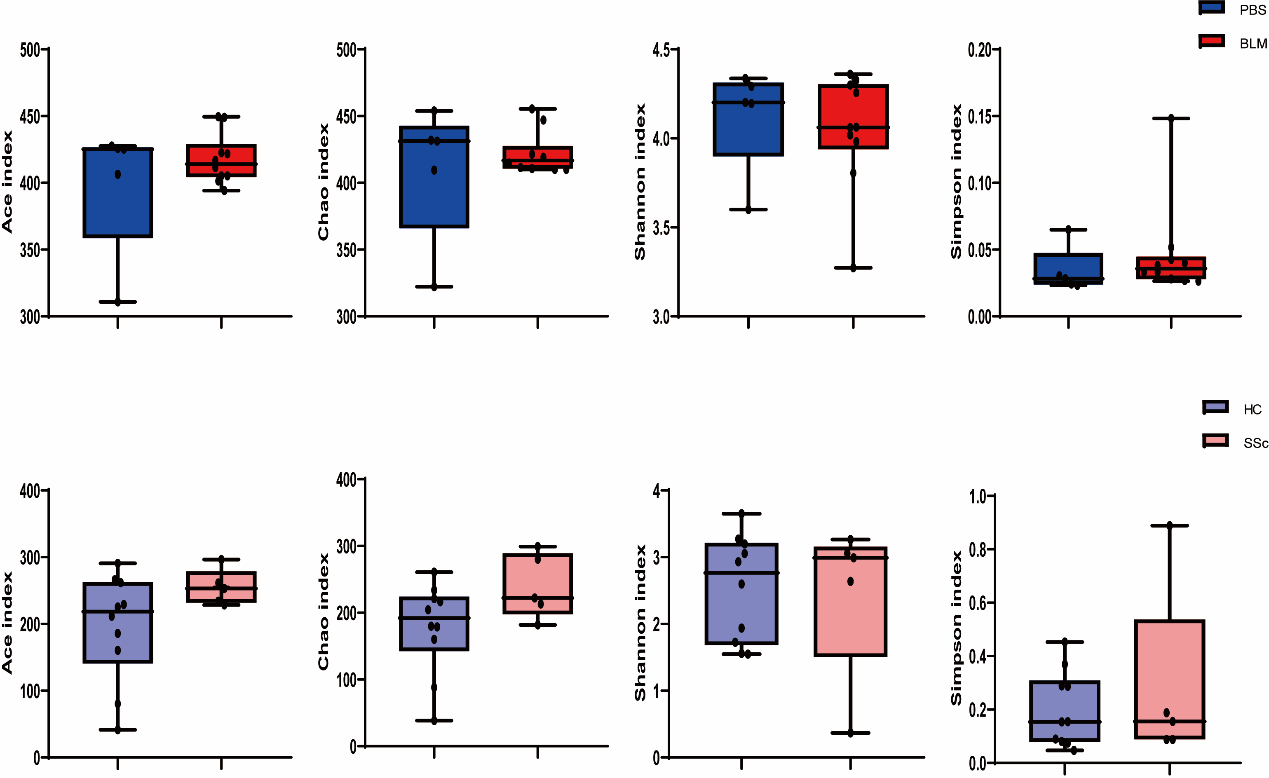


**Supplementary Figure S2:** Alpha diversity indexes in mice and human.There was no statistically significant difference in diversity between the two groups in either mice or humans.
